# Ultrastructure of *Sarcoptes scabiei* in Crusted Scabies

**DOI:** 10.4269/ajtmh.24-0279

**Published:** 2024-07-16

**Authors:** Yan Zhong, Xiu-Jiao Xia, Ze-Hu Liu

**Affiliations:** Department of Dermatology, Hangzhou Third People’s Hospital, Hangzhou, China

**Figure 1.**
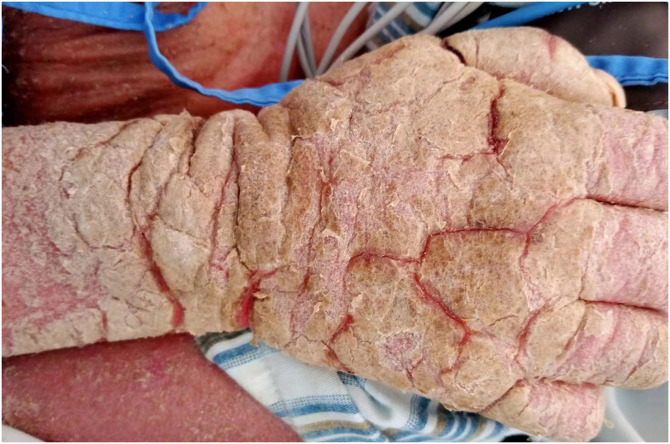
Diffuse erythematous hyperkeratotic plaques with fissuring in both arms.

**Figure 2.**
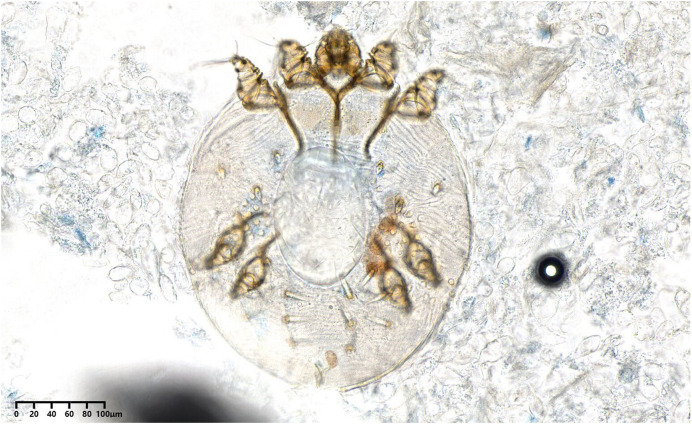
Direct microscopy shows adult mite with egg (×250).

**Figure 3.**
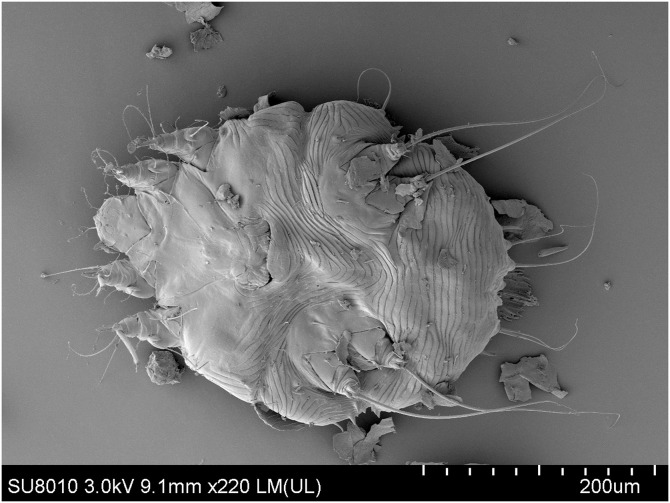
Scanning electron microscopy shows an adult female mite (×220).

**Figure 4.**
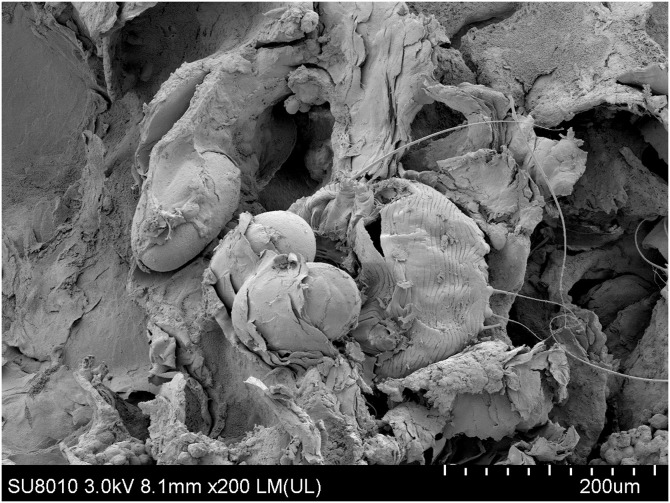
Scanning electron microscopy shows a fertilized female mite in a burrow with eggs (×220).

**Figure 5.**
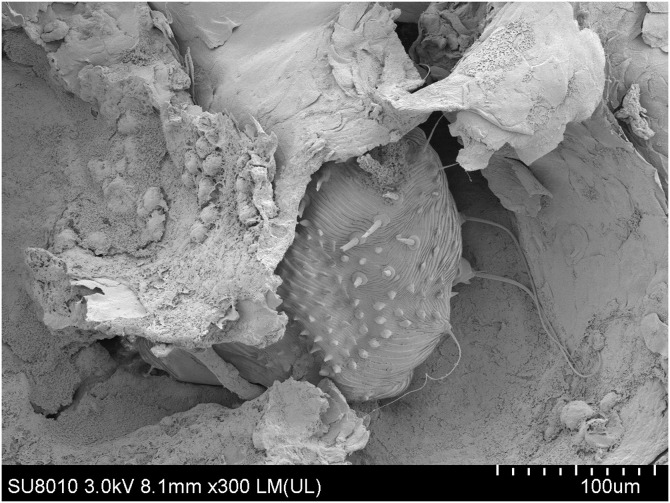
Scanning electron microscopy shows an adult female mite in a burrow and hardened fecal masses in a burrow (×300).

**Figure 6.**
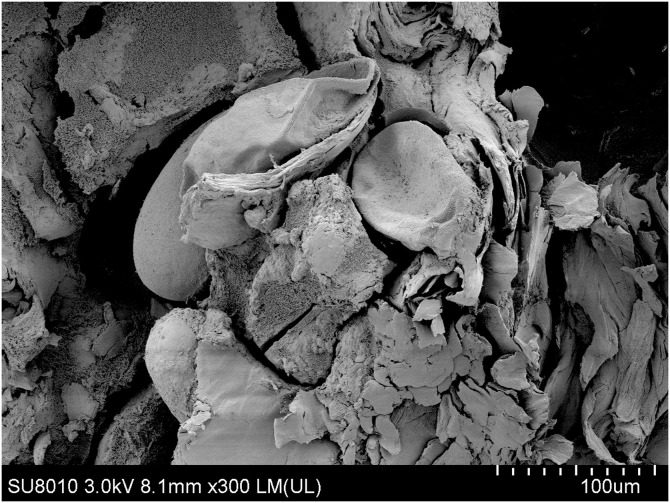
Scanning electron microscopy shows eggs with a larva within and postpartum eggshells (×300).

A 38-year-old woman presented with pruritic, erythematous, fissuring hyperkeratotic plaques involving the trunk and both arms for the previous 4 years. The disease worsened after topical use of glucorticosteroid cream prescribed for eczema. Her past medical history included epilepsy, schizophrenia, and polio when she was 6 years old. Diffuse erythema and scaling of the skin and hyperkeratotic, yellow-crusted lesions accompanied with fissures were observed on both hands (Figure 1). Her laboratory values were notable for an elevated eosinophilia (1.22 × 10^9^/L; normal <0.5 × 10^9^/L) and low total albumin (22 g/L; normal 40–55 g/L). A microscopic examination of scrapings from the hyperkeratotic lesions showed numerous *Sarcoptes scabiei* mites (Figure 2). A diagnosis of crusted scabies was made. Her husband also complained pruritus for more than 3 years. Scanning electron microscopy showed adult female mites (Figure 3), female mite with eggs (Figure 4), female mite and scybala (hardened fecal masses) in a burrow (Figure 5), eggs with a larva within, and postpartum eggshells (Figure 6). The patient was unsuccessfully treated with combo therapy including keratolytic solution (Chinese traditional medicine) and topical scabicides (10% precipitated sulfur petrolatum). The patient died of secondary sepsis 1 month later after diagnosis.

Crusted scabies is a relatively uncommon form of infestation with *S. scabiei* with generalized hyperkeratotic, crusted skin alteration and rarely evolves to erythroderma.^1^^,^^2^ Crusted scabies typically affects elderly, debilitated, immunocompromised, or long-term topical or systemic glucorticosteroid patients, the latter of which seems to have been the case with the present patient. A human with crusted scabies has innumerable mites with a high propensity to affect others who come into direct contact.^3^
